# Enhancing Mouth-Based Emotion Recognition Using Transfer Learning

**DOI:** 10.3390/s20185222

**Published:** 2020-09-13

**Authors:** Valentina Franzoni, Giulio Biondi, Damiano Perri, Osvaldo Gervasi

**Affiliations:** 1 Department of Mathematics and Computer Science, University of Perugia, 06123 Perugia, Italy; 2 Department of Mathematics and Computer Science, University of Florence, 50121 Firenze, Italy; giulio.biondi@unifi.it (G.B.); damiano.perri@unifi.it (D.P.)

**Keywords:** transfer learning, convolutional neural networks, emotion recognition

## Abstract

This work concludes the first study on mouth-based emotion recognition while adopting a transfer learning approach. Transfer learning results are paramount for mouth-based emotion emotion recognition, because few datasets are available, and most of them include emotional expressions simulated by actors, instead of adopting real-world categorisation. Using transfer learning, we can use fewer training data than training a whole network from scratch, and thus more efficiently fine-tune the network with emotional data and improve the convolutional neural network’s performance accuracy in the desired domain. The proposed approach aims at improving emotion recognition dynamically, taking into account not only new scenarios but also modified situations to the initial training phase, because the image of the mouth can be available even when the whole face is visible only in an unfavourable perspective. Typical applications include automated supervision of bedridden critical patients in a healthcare management environment, and portable applications supporting disabled users having difficulties in seeing or recognising facial emotions. This achievement takes advantage of previous preliminary works on mouth-based emotion recognition using deep-learning, and has the further benefit of having been tested and compared to a set of other networks using an extensive dataset for face-based emotion recognition, well known in the literature. The accuracy of mouth-based emotion recognition was also compared to the corresponding full-face emotion recognition; we found that the loss in accuracy is mostly compensated by consistent performance in the visual emotion recognition domain. We can, therefore, state that our method proves the importance of mouth detection in the complex process of emotion recognition.

## 1. Introduction

Visual emotion recognition (ER) has been widely studied as one of the first affective computing techniques, based on visual features of the face, to combine features about the eyes, mouth and various facial elements at the same time. Several different approaches to visual recognition obtained different grades of classifications for different visual recognition techniques [1,2,3]. Recently, studies using only the mouth for facial emotion recognition obtained promising results, while still not gaining the proper recognition among the state-of-the-art. Such works used convolutional neural networks (CNNs) to detect basic emotions from innovative and ubiquitous devices, e.g., smartphone or computer cameras, to produce textual, audio or visual feedback for humans, or digital outputs to support other services, mainly for healthcare systems [4]. A neural network can obtain an excellent result with a relatively small dataset of images when trained on a single individual, e.g., to detect particular states needing immediate medical intervention, or changes over time indicating an underlying degenerative health condition.

Our analysis focused on emotion classification with the mouth only, to study how much the mouth is involved in emotional expression and can provide accuracy compared to full-face recognition. We analysed the position and curve of lips through CNNs. Recently, some of our preliminary works [1,4,5] obtained favourable results regarding emotion analysis using the mouth, and this is our first attempt to recap and complete our full study on mouth-based emotion recognition with extensive datasets. All our previous works used convolutional neural networks to reach their goals on the topic, using a self-collected dataset. In-depth work has been primarily done on three emotions (i.e., joy, disgust and neutral) [5], among which disgust is the less studied in the literature.

After proving that the mouth can be itself a promising element for emotion recognition, in this work, we focus on the mouth as a unique element for facial-expression-based emotion recognition using advanced deep learning techniques. The goal of this approach was to enhance the mouth-based approach to emotion recognition in order to generalise the previous experimentation on a multiple-user dataset. To that end, advanced deep learning techniques have been implemented, i.e., knowledge transfer with elements of continuous learning. Such techniques are particularly suitable to being applied in the healthcare environment.

For instance, connecting this architecture to appropriate services can help users to convey emotions in an automated way effectively, e.g., providing augmented emotional stimuli to users affected by autism or other conditions involving social relationship abilities wherein a user experiences difficulties in recognising emotions expressed by other people. Another example may be a system able to recognise severe conditions and call a human assistant for intervention, e.g., for hospitalised patients feeling intense pain or needing psychological support. Such applications may provide feedback from healthcare personnel to exploit continuous learning.

We tested the most promising neural networks [1] for face recognition and mouth recognition, e.g., lip reading [6], with previous training on widely used large datasets of images [7]. Knowledge transfer allowed our neural networks to be pre-trained on low-level features, e.g., edges, corners and colour distribution.

In the last layers of the CNN, we carried out ad-hoc training dedicated to human face recognition of emotional classes. This work concluded the experiments by testing and comparing several CNNs on a widely-used dataset [8] of human faces labelled with emotions from the Ekman model. As in the preliminary work, for the final dataset we provide a filtered version, wherein photos showing simulated emotions, i.e., non-spontaneous expressions, have been removed.

### 1.1. Data Availability Issues for Facial ER

The main problem in facial emotion recognition is the lack of proper datasets of images for training. Most of the available datasets take into consideration only the Ekman model [9] or its subsets [5], discarding more complex and complete models, such as Plutchik [10], or models based on emotional affordance [11]. Moreover, image datasets often contain non-genuine expressions (e.g., simulated by professional actors) rather than spontaneous and natural forms of facial expression [12]. Since deep learning can extract features not even recognisable by humans, items related to non-real emotions make it hard to train a neural network effectively for emotion recognition. For these reasons, our work focused on a subset of the Ekman model, augmented with the neutral expression as a state of control for recognition results on emotions [4], by investing a fair amount of effort into selecting proper images from the available datasets, both from internet sources and from self-produced material. Collecting emotional images is feasible for emotions which are easily triggered, e.g., joy and disgust, but it is quite challenging for other emotions wherein ethical issues are involved in the stimulus, e.g., anger and fear. In order to improve results with a relatively small set of images for each emotion, we used transfer learning.

### 1.2. Previous Works

Previous works focused on the acquisition of data from a single user, i.e., where researchers train the network on a specific user’s face. This approach lets a user train the network with precision on his/her face, reaching advanced recognition performance [1,4]. Our work presents a more general approach using a multi-user dataset, containing images of users who were different regarding age, cultural background, gender and appearance. To that end, we used advanced methods of deep learning, i.e., transfer learning and continuous learning.

Multidisciplinary studies at the basis of our work in artificial intelligence (AI) stressed the importance of computer science for automated real-life tasks for assistive technologies [13,14]. Among them, labial detection and lip-reading [6] constitute our main background starting point [15].

One of the most promising advances of recent years for AI-assisted health care is the opportunity to develop mobile applications on widely-spread devices [4], e.g., smartphones, tablets and smartwatches, to support disabled users.

## 2. Problem Description and Proposed Solution

Our study exploits the high precision of CNNs by processing mouth images to recognise emotional states using the most recent advances in affective computing. Affective computing is a novel topic in artificial intelligence, defined for the first time in 2003 by Rosalind Picard [16], soon becoming one of the most trending multidisciplinary studies. Affective computing, involving the collaboration of several disciplines, e.g., psychology, physiology, neurology, liberal studies, computer science and robotics, recently stressed the importance of the extraction and recognition of affective mental states, e.g., emotions, moods, sentiments and personality traits. Notwithstanding the interest in such new research, most of the available research still focuses on trivial sentiment analysis or pure theoretical models (e.g., in psychology and social sciences) and product-based applications [17], mainly for marketing purposes. We prefer to focus on self-aid [18], health management and communication for understanding and supporting humans in real-life problems for any demanding task (e.g., due to disabilities [19], psychological states in emergencies or particular environments) with automated detectors and artificial assistants with machine emotional intelligence capabilities [20].

In our socially interconnected world, individuals already use and produce daily an overwhelming amount of heterogeneous data in a manageable and personalised subset of classified items. Data can be directly used for emotion recognition (ER), e.g., photos and text shared on social networks; or can contain elements indirectly inferable for emotional intelligence, such as physiological data collected by wearables—including sleep patterns, continuous heart-rate and movement tracking, emotions expressed by art [21] and data from users experiencing exciting games [22]. If sentiment analysis relates only to recognising the positiveness, negativeness or neutrality of sentiments, moods and emotions, the process of emotion recognition is still little studied, implying the recognition of specific emotions of an emotional model. Since scientists do not agree on all the available models, research on ER in any applicative domain starts from the widely recognised model of Ekman, which we chose for our study. Recent research underlines that Ekman’s primary emotional states, including happiness, sadness, anger, disgust and neutral [23] can be recognised based on text [24] and physiological clues such as heart rate, skin conductance, gestures, facial expression and sound, which can be managed with a multidimensional approach [11] and compared.

Among all ER approaches, facial recognition is still predominant. Under that point of view, we decided to focus on the mouth as a particular facial element, almost always visible in any facial expression, even considering that some cultures underestimate the mouth’s expressiveness more than others. Some tribes in South America, for instance, as Ekman himself underlined in his first experiments on facial emotions labelling [9], rely more on the upper part of the face. The new issue is that, in general, when comparing such original studies with our results in AI, the neutral emotion seems the most misunderstood both by humans and artificial agents; it tends to be labelled as anger or sadness. The fact that the same emotion is also the most mistaken by automatic mouth-based recognisers in the same classes of errors confirms that the automatic agent can recognise images correctly, based on human labels.

In this work, we tested the technique on multi-user datasets, to find solutions to the following research questions:

- With how much precision it is possible to recognise facial emotions solely from the mouth?
- Is the proposed technique capable of recognising emotions if trained on a generalised set of facial images?

In a user-centred implementation, the software supports personalised emotional feedback for each particular user: personal traits, such as scars or flaws, and individual variations in emotional feeling and expression, help the training to precise recognition. The system can recognise different users because it was trained on a comprehensive dataset, including images varying in ethnicity, age and gender.

In order to obtain optimised results, the ambient light setting should not require a particular setup. A consistent implementation should meet the following requirements:

- Robustnes. The algorithm must be able to operate even in the presence of low-quality data (e.g., low resolution or bad light conditions);
- Scalability. The user’s position should not be necessarily fixed in front of the camera, in order to avoid constraining the person. Therefore, the software should be able to recognise the user despite the shot’s point of view;
- Luminosity. Luminosity is an important issue because the variation of light hardly influences the recognition capabilities of a CNN-based automated system. Available datasets usually provide photos shot under a precise lighting setup. On the contrary, a sufficient number of training samples of each considered lighting (e.g., natural light, artificial bulbs and low-light conditions) should be provided for an efficient categorisation.

Our implementation of mouth-based ER exploits transfer learning (i.e., knowledge transfer), which uses general-purpose neural networks pre-trained on extensive datasets including different shapes, later fine-tuned on the classification domain (i.e., mouth-based emotion recognition). Our application can be easily adapted for continuous learning, given a domain where said method is useful, and appropriate data are available as feedback to track the weights of the neural network and prosecute the training. Such a method should allow enhancing the precision of emotion recognition in real-time situations, where the final weights of a previous training can be used in a subsequent time. The collateral effect of such a technique is the requirement of more exceptional computational capabilities and a semi-supervised system in order to stop and roll back in cases of glaring errors or overfitting in a specific environment, e.g., a particular camera resolution or light situation in a specific place or with certain timing. Continuous learning can continuously adapt and enhance the network’s accuracy, leading on the go to a more reliable system, when used for a prolonged time.

## 3. The Framework Implementation

The proposed implementation has been developed using the Python programming language and the Keras framework [25]. The tests were carried out using the Google Colab platform, in which the Python code was developed and executed, exploiting the powerfulness of the Keras libraries. Data analysis and processing have been developed with a chain of operations. The implemented procedure can be described with the following steps:

1. Raw dataset import. In the first phase, the dataset (at the beginning, a raw dataset) is composed of RGB images and is imported into the system.
2. Raw dataset cleaning. All the faces in the photos of the dataset are manually scanned, and the images representing fake emotions (e.g., simulated by professional actors) are discarded.
3. Data set generation. From each image of the raw dataset, the portion representing the mouth of the subject is automatically extracted.
4. Training with data augmentation. At every step of the training, the system reads a partition of images from the dataset. At each reading, the system performs data augmentation [26] using random values of rotation degree within a given range. The model used for image analysis is exploited using transfer learning.
5. Model generation. At the end of the training, the structure and weights of the neurons that achieved the best performance in training are saved.
6. Results Evaluation. To evaluate the quality of the model, we analyse the accuracy of ER on the validation set.

The mouth extraction from the images of the raw dataset was carried out using as a pre-trained neural network the shape_predictor_68_face_landmarks.dat CNN [2,8,27], which produced in output 68 landmarks, detected for each image. The shape predictor was pre-trained on the ibug 300-W dataset. The landmarks are expressed as a series of coordinates identifying specific elements of a face, e.g., the positions of the mouth, eyes and cheekbones. Once we obtained the landmarks of a face, we used those identifying the area of the mouth and cropped the image (i.e., cutting out the original image to obtain only the part related to the mouth). All images of the mouth were transformed in size to 299 × 299 pixels.

**Figure 1:**
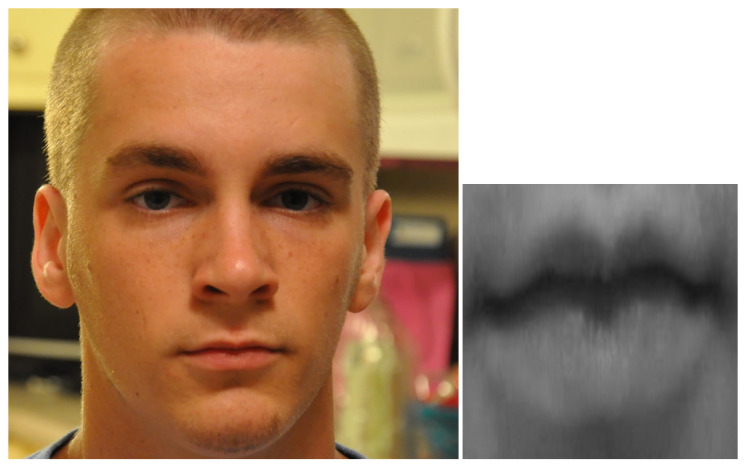
Mouth detection, cropping and resizing (source image from AffectNet database).

In Figure 1, an example of mouth detection, cropping and resizing is shown. On the left side, an image taken from the dataset is visible; on the right, the corresponding extracted mouth. At the end of the process, the mouth images were stored in a directory structure associated with the labelled emotion; a final inspection of the mouth images dataset shows that the mouth is always correctly cut, whether it is open or not. We used data augmentation techniques [26] to increase the robustness of the neural network, thereby randomly transforming the images, i.e., with a flip and a random rotation within the [−4, +4] degree range.

### 3.1. Convolutional Neural Networks

Convolutional neural networks constitute a class of deep neural networks which prove particularly efficient for different tasks on data organised in a grid topology, e.g., time series and visual inputs, and are among the most used for deep learning in image-based classification. Typical applications include image recognition, segmentation, detection and retrieval [28,29,30], on which CNNs achieved state-of-the-art performances. This significant breakthrough can be attributed to three critical factors boosted by CNNs, i.e., sparse interactions, parameter sharing and equivariant representation [31]; massive amounts of training samples can be processed with improved efficiency and significantly reduce training times, using deeper networks with millions of parameters to learn more complex and characteristic image features. An additional advantage is the possibility of having a variable input size, in contrast with traditional, fully-connected networks which require fixed-size input. In convolutional neural networks, some of the traditional fully-connected layers are replaced with convolution layers, which scan through the ordered, grid-like structured data to process the data in subsets, and multiply each subset by a kernel matrix (i.e., filter) to produce a feature map in output. The process resembles how individual neurons respond to visual inputs: each neuron is responsible for a small portion of the input, called its receptive field, and ignores additional information. Training a fully-connected neural network with the same feature recognition, and consequently, classification capabilities, would require much greater effort—e.g., for an image of size 1000 × 1000, training 1,000,000 weights for each neuron of a layer. In contrast, CNNs would have some trainable parameters dependent on the size of the applied kernel, but still in a much lower amount.

Typically, CNNs are interlaced sequences of three different types of layers: the previously described convolution layers, conventional fully-connected layers and pooling layers, used to aggregate multiple features into a single one, i.e., down-sampling the feature maps according to different strategies. The CNNs used in literature for reference tasks such as ImageNet classification, although varying in the number of layers, filter size and quantity, are mostly composed by the building blocks cited above.

### 3.2. CNN Settings

The experiments have been performed on four convolutional neural networks: VGG16 [32], InceptionResNetV2 [33], InceptionV3 [34] and Xception [35]. Among CNNs, these networks outperform AlexNet [28] on the widely used ImageNet [7] dataset, which is one of the largest image datasets used for the transfer learning pre-training phase.

**Figure 2:**
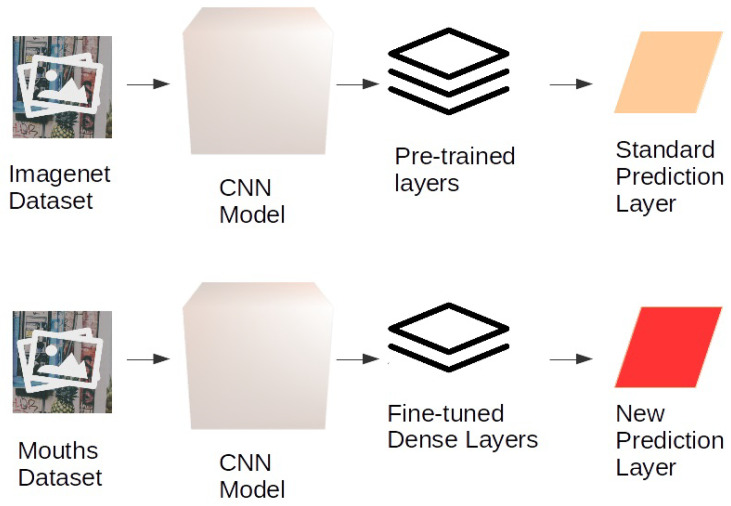
General scheme of the adapted transfer learning techniques with the considered CNNs.

**Figure 3:**
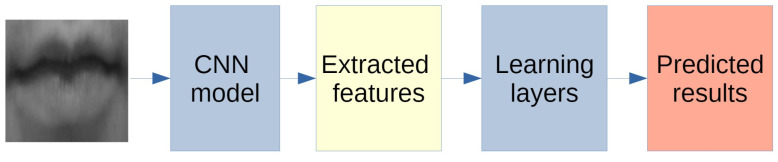
General scheme of our CNN.

All neural networks, whose general behaviour is described in Figure 2, have been tested using transfer learning (see Figure 3 showing the process for a general CNN).

Adopting transfer learning, we used the pre-trained neural networks, whose weights have been computed on the ImageNet [7] dataset. The first layers of the networks have been frozen for the training phase; i.e., the weights of the convolutional layers have been fixed and have not been altered during the fine-tuning phase on the mouth emotion dataset. This was done due to the high capability of the CNNs to recognise low-level features, e.g., points, lines, edges, corners and colour distribution. We replaced the final layers to fine-tune the network on mouth emotion recognition, using two dense layers with 64 neurons each and a SoftMax layer. This final layer ranks the likelihood of the most appropriate class, thereby returning the emotion classification. Only the final layers of the CNN networks, i.e., the fully-connected and the final SoftMax level, therefore, change and can be re-trained in the fine-tuning phase. The model was set up to save the weights only when they improve the emotion classification accuracy concerning the previous epoch, resulting in a final best neural network training configuration.

We tested two optimisers: Adam and SGD. Adam performed better with InceptionV3 and VGG16 with a learning rate equal to 0.001; and with Xception it had a learning rate of 0.01. SGD performed better with InceptionResNetV2 with a learning rate equal to 0.001, momentum = 0.9 and nesterov equal to true. The remaining parameters used were the following. Batch size equal to 25 and the maximum number of epochs equal to 100; however, we used the early stopping technique: if results do not improve, the training is stopped.

## 4. Data Set and Filtering Phase

As introduced in Section 1.1, in the actual state-of-the-art, one of the most relevant issues for our goal is the total lack of a sufficient number of images of real emotions. Most of the available datasets, in fact, include a mixed set of real and fake emotions, performed by actors or pretended, which cannot be considered usable for a technique such as deep learning, which is able to base the classification on very detailed features, which are different between real and fake emotions even at a micro-expression level, and may vary in different cultures. A person who is striking a pose for a photograph (e.g., smiling voluntarily in front of the camera) often assumes an artificial, distorted expression that is not generated autonomously by the human brain, even if it can be appropriately identified and recognised by the human brain as a smile, thanks to mirror neurons. We must report that most of the available datasets provide images depicting actors; thus, a cleaning phase of the chosen dataset was mandatory. It should be considered that there are emotions such as anger and fear that are not easily replicated in a laboratory, whereas emotions such as joy or the neutral state, on the other hand, can be easily triggered.

Another relevant limitation is that many datasets include only images of a particular light state, perspective and image resolution, which leads to the system overfitting that particular environment and failing in others. Until a proper extensive dataset is ready for the test, all results have to be considered preliminary.

**Figure 4:**
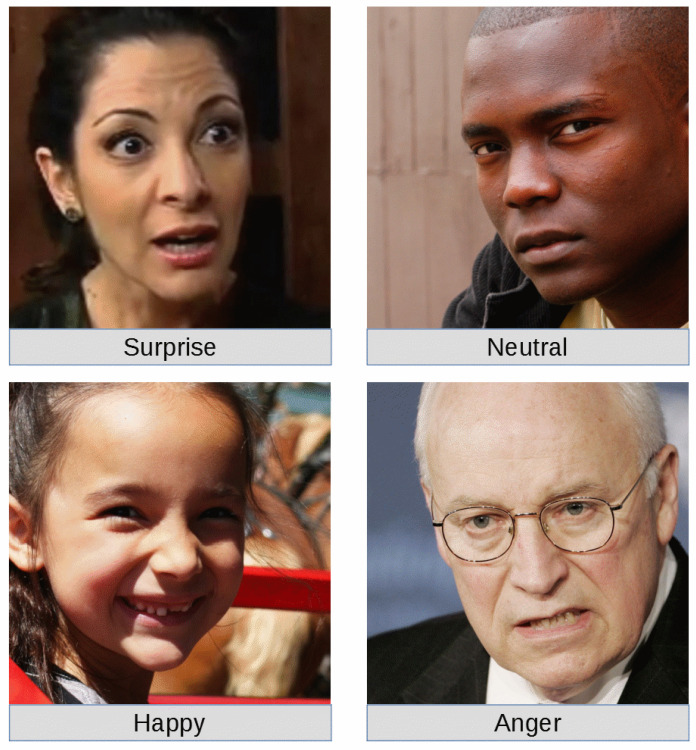
Sample images from the filter dataset.

For the emotional training (i.e., fine-tuning of the CNNs) and classification test of our work, the dataset AffectNet provided by the University of Denver [36], USA, has been used. The raw dataset is composed of the images of a large number of faces, and a spreadsheet file used to assign to each image an emotion label. We analysed a subset of the Ekman emotions, i.e., neutral, happy, surprise and anger. A sample image for each class of emotion is shown in Figure 4.

The dataset has been cleaned by analysing images and removing duplicates. The automated mouth recognition removed also all the items where the mouth was not visible. Though grainy or unsatisfactory resolution photographs can be removed, it is also essential to maintain significant differences in resolution, point of view and light conditions. This variety helps the network to train on general images and not on only those with particular light conditions, resolution and so on.

The primary filtering improvement of the dataset has exploited the contents of images, while avoiding all the photographs showing fake emotions, i.e., facial expressions apparently simulated.

In Table 1, the number of images per type of emotion in the cleaned dataset is shown.

## 5. Results and Discussion

The experimental work has been divided into two phases. A preliminary phase included experiments involving freezing the pre-trained convolutional layers and fine-tuning the fully-connected layers, as explained in Section 3. The results suggest discarding the CNN networks with the lowest accuracy percentages, i.e., VGG16, Inception V3 and Xception, while keeping InceptionResnetV2, which achieved the best performance. In the second phase, the freezing of InceptionResnetV2 was removed to further fine-tune all the layers of the network. For each network, the best model obtained in the training phase was saved, and we rolled back the weights to keep the highest accuracy. Iterating this process, we obtained a final accuracy of 79.5% on the validation set using the best network, as shown in Table 2. As shown, the network that performed worst was VGG16, while the best network was InceptionResnetV2; in Table 2, we included the final results for all the networks. Note that only InceptionResnetV2 has been fine-tuned in all its layers.

**Figure 5:**
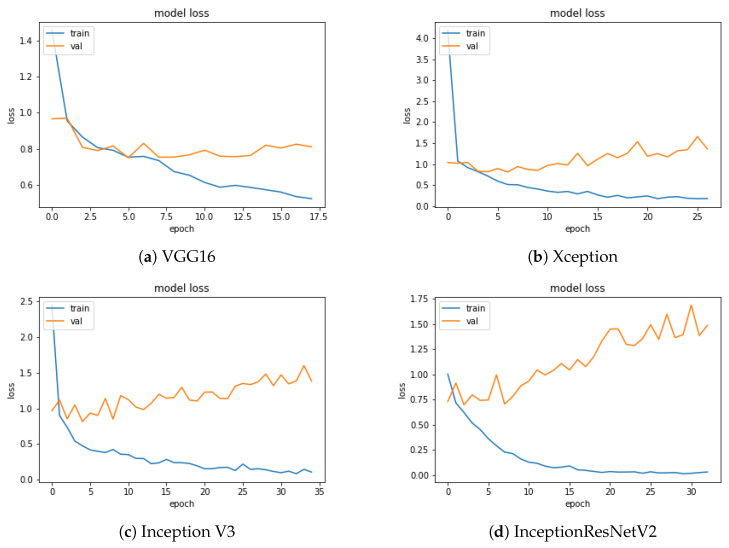
Loss function evolution for each network as a function of the epochs.

**Figure 6:**
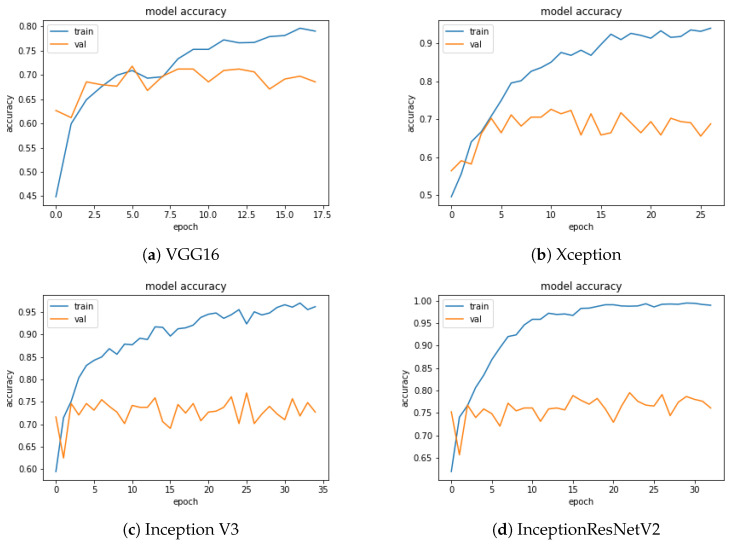
Accuracy function evolution for each network as a function of the epochs.

Figure 5 and Figure 6 show the loss and accuracy values of the model as a function of the training epochs. The number of epochs was different for each CNN, due to the use of the early stopping criterion described in Section 3.1. Early stopping is essential to eliminating or mitigating the overfitting effect that, as the epochs grow, would lead the accuracy on the training and validation sets, represented by the two curves (blue and orange), to diverge due to a loss of generalisation capability of the trained model. In agreement with the accuracy, InceptionResNetV2 shows the best trend for the loss function.

In Figure 6 we can observe for the InceptionResNetV2 a higher accuracy and a more regular shape of the function, compared to the other CNNs.

**Figure 7:**
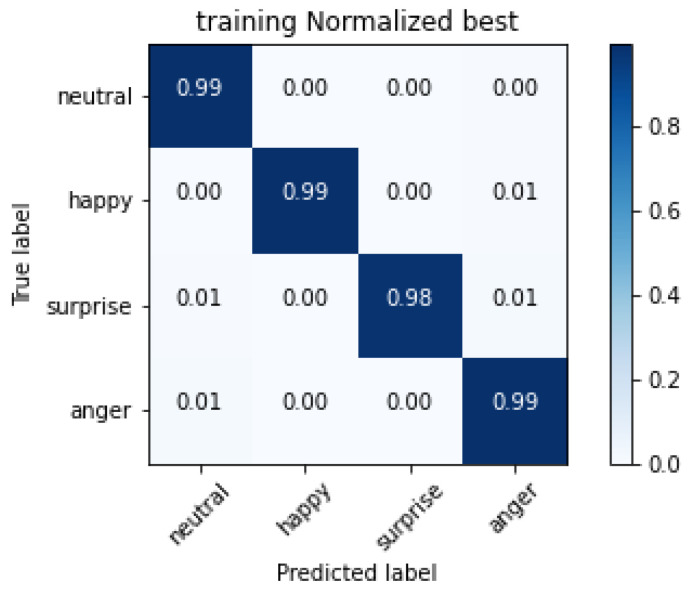
Confusion matrix of the training set for the network InceptionResNetV2.

The confusion matrix of the training set for the InceptionResNetV2 network is reported in Figure 7.

The cells on the diagonal of the matrix shown in Figure 7, representing correctly classified images, show good performances, close to the perfect classification. Errors appear in classifying happiness, sometimes interpreted as anger; the same issues occur for surprise interpreted as neutral and anger. Finally, anger is sometimes misinterpreted as neutral. As introduced in Section 2, the misclassification of the neutral emotion presents a different issue, thereby requiring a separate discussion. Studies in cultural anthropology, including the first work of Paul Ekman on facial emotion recognition [9], show that humans sometimes tend to misclassify neutral as anger or sadness, with different results in different cultures. Such bias is clearly reflected in the AffectNet dataset because it is present in our automated recognition too. We can realistically suppose that our CNN correctly classified the mouths based on biased labels. In both the classification directions, neutral is sometimes misclassified, and the other classes are misclassified as neutral. In Table 3 the absolute values of misclassified images are reported for each class in the neutral evaluation. We can say that our evidence suggests that our networks behave similarly to the human brain, possibly learning the features associated with the human bias present in the considered dataset.

**Figure 8:**
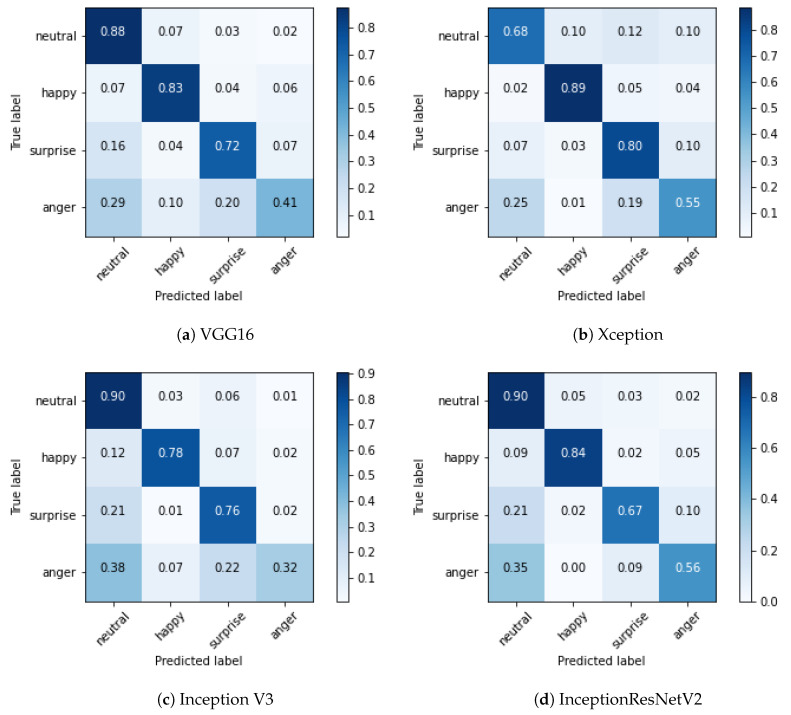
Confusion matrices of the trained networks, with normalised values.

The confusion matrix of the validation set relative to each tested network is shown in Figure 8.

**Figure 9:**
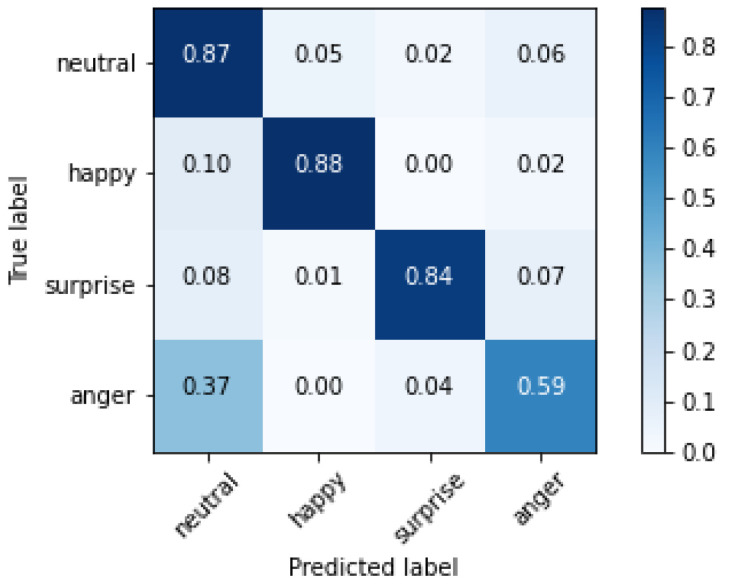
Confusion matrix of the validation set obtained while running InceptionResNetV2 to analyse the whole face images instead of the mouth portions of the images.

The experiments have been replicated on images of the whole face, to study the contribution of the mouth; the resulting confusion matrix is shown in Figure 9.

Results can be summarised in the following points:

- The network that provided the best performance was the same as the one for mouth-based ER, i.e., InceptionResNetV2.
- Anger was again the most difficult emotion to classify.
- The performance improvement was only 5%, with an accuracy rate of 84.4%, thereby proving the consistent contribution of the mouth for ER.

## 6. Conclusions and Future Developments

In this work, we presented the first consolidated results for the approach of mouth-based emotion recognition, gotten through the comparative analysis of four different CNNs using transfer learning.

We confirmed the significance of the mouth to classifying the user’s emotions and provided inspiring and useful information to understand its contribution compared to full-face recognition.

As described in Section 5 we obtained with the InceptionResNetV2 neural network an accuracy of 79.5% on the validation set. With respect to the full face, results show a loss of accuracy of only 5%, which in our opinion is offset by the advantages of our method. The mouth is, in fact, a critical element of human face recognition, almost symmetric and usually visible from any perspective, and thus the ideal element to focus on in all those cases wherein the user can be shot from any point of view. Moreover, focusing on a smaller area of the image requires lesser computational capabilities.

Straightforward applications of our approach are emotion/pain recognition for healthcare management, and automated supervision of critical patients, e.g., those bedridden in hospitals. The system can support patients by using an ER system when direct human assistance is not available, e.g., during the night or in wards where assistance is not allowed, for advanced detection of an initial pained or discomfort state, and raising a signal and letting the sanitary staff be informed to react promptly, thereby avoiding the patient’s suffering. Supportive systems can be planned using emotion recognition, e.g., to assist patients after car accidents, still in the emergency phase or post-surgery, in order to understand their pain levels. If the system recognises a critical situation, it can report the case to nurses or send an intervention/checkout request for the patient’s room. Our approach could be easily extended to the scenario previously described, upon the availability of proper datasets of pain images. An additional application could be the early recognition of depressive states or the support of people with difficulties seeing or interpreting emotions, e.g., blind users, or people with autism spectrum disorders. Our emotion recognition system should in future give feedback to the user (e.g., text, emoticon, sound), or we could set up a channel for information transfer to software.

## Figures and Tables

**Table 1:** Number of images per type of emotion considered in our study.

	Neutral	Happy	Surprise	Anger
*# of images*	1239	562	463	478

**Table 2:** Final results related to the considered CNNs.

Network	Accuracy
Vgg-16	71.8%
InceptionResNetV2	79.5%
Inception V3	77.0%
Xception	75.5%

**Table 3:** Number of misclassified images of neutral faces in the three wrong categories over a total of 221 images.

	Happy	Surprise	Anger
*# of images*	11	7	4
